# An Ovine Model for Percutaneous Pulmonary Artery Laser Denervation: Perivascular Innervation and Ablation Lesion Characteristics

**DOI:** 10.3390/ijms22168788

**Published:** 2021-08-16

**Authors:** Heber Ivan Condori Leandro, Elena G. Koshevaya, Lubov B. Mitrofanova, Aleksandr D. Vakhrushev, Natalia S. Goncharova, Lev E. Korobchenko, Elizaveta M. Andreeva, Dmitry S. Lebedev, Evgeny N. Mikhaylov

**Affiliations:** Almazov National Medical Research Centre, 2 Akkuratova Str., 197341 Saint-Petersburg, Russia; egk995@gmail.com (E.G.K.); lubamitr@yandex.ru (L.B.M.); advakhrushev@gmail.com (A.D.V.); ns.goncharova@gmail.com (N.S.G.); lev.korobchenko@gmail.com (L.E.K.); elizaa.andreeva@gmail.com (E.M.A.); lebedevdmitry@mail.ru (D.S.L.)

**Keywords:** pulmonary hypertension, pulmonary artery denervation, laser ablation, S100 immunolabeling, animal model

## Abstract

Background: Pulmonary artery denervation (PADN) is an evolving interventional procedure capable to reduce pulmonary artery (PA) pressure. We aimed to compare PA nerve distribution in different specimens and assess the feasibility of an ovine model for a denervation procedure and evaluate the acute changes induced by laser energy. Methods: The experiment was divided into two phases: (1) the analysis of PA nerve distribution in sheep, pigs, and humans using histological and immunochemical methods; (2) fiberoptic PADN in sheep and postmortem laser lesion characteristics. Results: PA nerve density and distribution in sheep differ from humans, although pigs and sheep share similar characteristics, nerve fibers are observed in the media layer, adventitia, and perivascular tissue in sheep. Necrosis of the intima and focal hemorrhages within the media, adventitia, and perivascular adipose tissue were evidenced post laser PADN. Among the identified lesions, 40% reached adventitia and could be classified as effective for PADN. The use of 20 W ablation energy was safer and 30 W-ablation led to collateral organ damage. Conclusions: An ovine model is suitable for PADN procedures; however, nerve distribution in the PA bifurcation and main branches differ from human PA innervation. Laser ablation can be safely used for PADN procedures.

## 1. Introduction

Pulmonary hypertension (PH) is a progressive disease with a malignant prognosis [1]. PH pharmacological therapy has been shown to improve patient hemodynamic status and quality of life [2]. However, novel non-pharmacological modalities are evolving. Pulmonary artery denervation (PADN) is a surgical or interventional procedure aiming to modulate pulmonary artery (PA) pressure in patients with pulmonary arterial hypertension (PAH) of different etiologies [3,4,5,6]. The abatement of perivascular nerves within and around the pulmonary artery (PA) resulting in the decrease of sympathetic neural activity has been suggested as a pathophysiological consequence of PADN procedures [7]. Recently, PA stimulation mapping delineating the phrenic and laryngeal recurrent nerves close to the PA has been demonstrated to be feasible during transcatheter PADN. Moreover, evoked reactions during high-frequency stimulation reveal specific autonomic responses in determined areas, which might be potential targets for PADN [8].

PADN strategies are still not well defined, and radiofrequency energy has been the first energy source employed for PADN in PH subjects and followed by the application of ultrasound energy [3,9]. Furthermore, the recently developed approaches utilized fluoroscopy guidance, and PA three-dimensional electroanatomical reconstruction has been used in a minority of studies [5,7,10]. Technology evolution allowed the manufacture of optical fibers for laser devices and consequently the development of intravascular and intracardiac catheters for medical interventions. Despite the wide applications of lasers across various therapeutic and diagnostic fields, the effects of laser ablation on the PAs vasculature and perivascular nerves remain unknown.

Although different animal models have been suggested for the evaluation of PADN procedures, the large animal anatomy seems to better represent catheter maneuvers within the vasculature and heart. However, perivascular nerve distribution and, therefore, denervation results may vary between different specimens. The majority of experimental PADN studies utilizing radiofrequency energy have adopted a porcine model for their purposes [11,12]. We suggest that ovine vasculature would be appropriate as well, if not better, for laser denervation, since light absorption characteristics vary between specimens and might be better selected for experimental studies.

In the present experimental study, we aimed to compare ovine, porcine, and human PA nerve distributions to assess the applicability of an ovine model for the PADN procedure; and to assess the morphological and histological acute changes induced by laser catheter ablation on ovine PA wall and vascular and perivascular nerves.

## 2. Results

### 2.1. Pulmonary Artery Nerve Distribution in Experimental Large Animals and Humans

Differences in nerve distributions among PA wall layers between three specimens were noted. In sheep, nerve fibers were located in the perivascular adipose tissue, adventitia, and, starting from the PA bifurcation level, nerves penetrated media in all cases. The porcine PA wall was characterized by innervation of the perivascular adipose tissue and adventitia. In human PA preparations, nerve fibers were only found in the perivascular adipose tissue in all the studied clusters: the PA trunk, bifurcation, and main branches.

**Figure 1:**
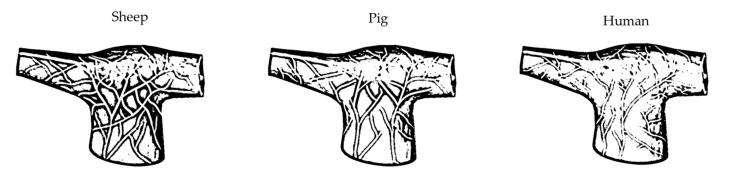
A schematic representation of nerve distribution of the pulmonary artery in different specimens.

The nerve diameter and nerve distributions in porcine and ovine were significantly different from those in human PA preparations (Table 1). Thus, porcine and ovine nerves were much thinner than human nerves on all levels. At the same time, the density of nerve fibers was significantly higher in animals when compared with humans (Figure 1).

The mean depth of nerves was statistically similar between the specimens at the level of the PA trunk. While more distally, nerves were farther from the intima in human PA preparations than in ovine and porcine preparations, reflecting the lack of PA wall penetration.

Generally, ovine PA was characterized by similar to porcine nerve diameter and density, and a similar mean distance from the PA lumen to nerve fibers.

Within each specimen, the mean nerve fiber diameter was comparable at the levels of PA trunk, bifurcation, and main branches: for ovine PA *p* = 0.912, porcine *p* = 0.092, and human *p* = 1.0; the mean nerve density was also comparable at different levels within specimens (*p* = 0.804; 0.359; 1.0, correspondingly). The mean nerve fiber depth in the PA trunk and bifurcation sections varied significantly in sheep and was located deeper in the adipose tissue (*p* < 0.001), unlike in humans and pigs (*p* = 0.98; 0.320).

### 2.2. Ovine Pulmonary Artery Laser Denervation and Lesion Histological Evaluation

**Figure 2:**
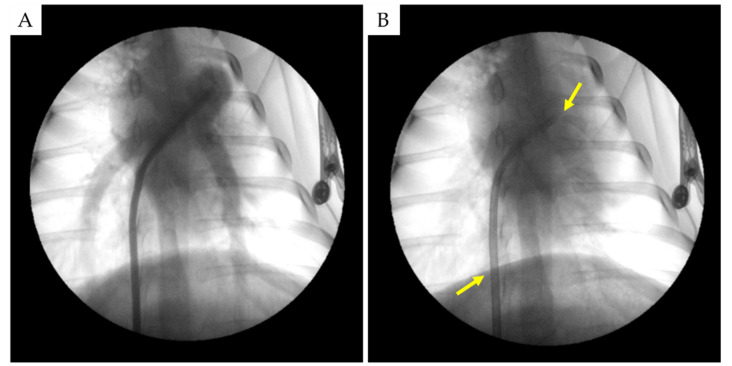
(**A**) Pulmonary artery angiography in sheep №1. (**B**) The laser catheter (arrow) is placed through the steerable sheath (arrow) in the PA trunk. Sheep №1.

A total of 42 ablation sessions were performed: around the PA trunk (*n* = 12), bifurcation (*n* = 7), and the proximal areas of the left (*n* = 11) and right (*n* = 12) PAs (Figure 2A,B). Detailed ablation characteristics are shown in Table 2.

#### 2.2.1. Necropsy Findings

**Figure 3:**
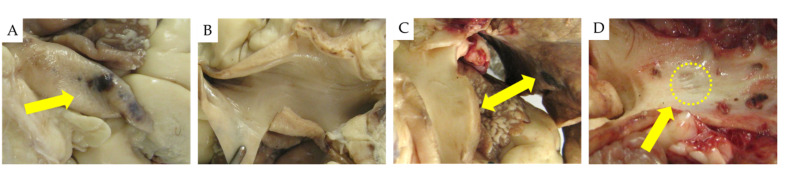
(**A**) Hemorrhagic lesion observed in the adventitia of the PA trunk post laser denervation in Sheep №1; 20 W, 20 s ablation. (**B**) No lesions are visible on the intima of the left and right PA branches in Sheep №2; 30 W, 20 s ablation. (**C**) Hemorrhagic collateral damage in the left lobe lung in Sheep №2; 30 W, 20 s ablation. (**D**) A rough defect is observed in the right PA in Sheep №3; 20 W, 35 s ablation.

Fifteen lesions were visually identified during PA gross anatomical evaluation. The lesions observed in the PA were inhomogeneous. In sheep №1, evident laser-related lesions described as irregular brown hemorrhage spots were observed in the PA trunk (Figure 3A); in Sheep №2, no lesions were observed during gross anatomy evaluation before fixation (Figure 3B); and in Sheep №3, rough defects and lung collateral lesions were evident (Figure 3C,D).

After fixation in formalin, 10 additional ablation spots were identified on the PA surface: 2 in sheep №1, and 8 in sheep №2. In total, 25 ablation spots were identified and evaluated microscopically.

#### 2.2.2. Histological and Immunohistochemical Changes Post PADN Using Laser Energy

**Figure 4:**
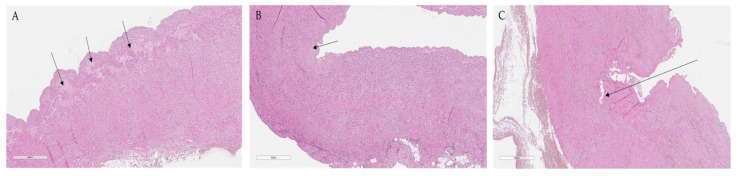
(**A**) Grade I lesion-focal edema (arrows) of the inner third of the media and extending to a depth of up to 10% of the thickness of the media, ×50. (**B**) Grade II lesion-focal necrosis of the intima and the inner third of the media (arrow) and depth up to 10% of the thickness of the media layer, ×50. (**C**) Grade III lesion-dissection of the artery wall with a depth of more to 50% of the media thickness (arrow) and hemorrhages at any depth of the media, in the adventitia and adipose tissue (with a diameter more than 800 µm), ×50.

**Figure 5:**
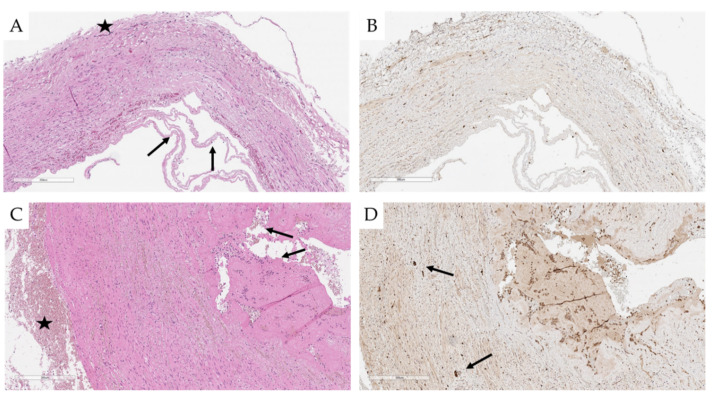
(**A**) A section of the pulmonary artery with dissection and hemorrhage (an arrow-intima with necrosis, a star adventitia), ×100 (Sheep №1). (**B**) A similar section area with S100 immunolabeling, ×100 (Sheep №1). (**C**) Dissection of the pulmonary artery trunk, fibrin thrombus at the site of dissection (arrow), hemorrhage in the adventitia (shown by a star), ×100 (Sheep №2). (**D**) A similar section site with preserved S100 expression on nerve fibers (arrow) in media close to the dissection area, S100, ×100 (Sheep №2).

According to the microscopic findings on PA wall lesions and perivascular nerve injury, a semiquantitative grading of each detected laser ablation spot was performed (Table 3; Figure 4A–C). Laser PA ablation resulted in focal necrosis of the intima and the underlying layer of the media, focal hemorrhages within the media, adventitia, and perivascular adipose tissue. In two out of three sheep, PA wall dissections in the PA trunk and right PAs were found (Figure 5A–C). Across the dissection areas, nerve fibers were not identified through the PA wall layers, and no specific staining was observed using the S100 marker (Figure 5B). In general, no direct damage to nerve fibers near the dissection sites was identified, and S100 expression was preserved (Figure 5D).

According to the lesion grading, 8 (32%) ablation spots had grade I lesion, 7 (28%) spots had a grade II lesion, and 10 (40%) spots had a grade III lesion. Therefore, 40% of the identified lesions reached the depth of adventitia nerves and could be considered effective for PADN. Table 4 represents the distribution of graded lesions resulting from different ablation power.

#### 2.2.3. Laser Lesion Safety

During the experimental procedure, all animals were stable and were euthanized as mentioned above. According to the presence of superficial disruption of PA layers and the presence of extra-PA structure damage (lungs and/or myocardium), the ablation lesions were conditionally defined as safe or unsafe (Table 5). 

Therefore, safe lesions were considered in 22 (88%) cases, and unsafe lesions in 3 (12%) cases, out of the 25 identified. Ablation with 20 W in 1 case and 30 W in 2 cases resulted in unsafe lesions and can induce collateral damage in the surrounding tissue. On the other hand, ablation using 15–20 W resulted in 22 identified safe lesion and seemed to be safer for denervation procedures. Additionally, 14 laser ablation sites with ≤20 W and 3 sites with 30 W power were not identified postmortem and, theoretically, could be classified as safe.

## 3. Discussion

The major finding of our study is that the ovine model can be used for PADN procedures when technical and instrumentational issues are evaluated. Specific catheter instrumentations dedicated for human use are easily adopted in ovine experiments on PA. Another important finding is that ovine PA nerve density and distribution significantly differ from those in humans. Thus, nerve density in the PA trunk, bifurcation, and main PA branches are much lower than in the human PA. This is very comparable to what is found in the porcine PA. Sheep is characterized by a specific feature: nerves penetrate the PA wall in the bifurcation and distally. Therefore, we suggest that the ovine model of PADN should be used with precautions when perivascular nerve damage is analyzed since the required lesion depth in sheep PA is much lesser than in the human PA to involve the majority of neural structures. Theoretically, hemodynamic consequences of PADN in sheep might be different from that in humans, since nerve density in the ablation area is significantly higher. The pulmonary vessels are innervated by subsets of sympathetic, parasympathetic, and sensory nerve fibers. Although most organs have a maximal neural density at distal levels, the pulmonary vasculature has an opposite pattern—the nerve density is highest at large-caliber vessels with a further decrease at the periphery [13]. In large mammalians, frequently used for pulmonary research models, the distribution of autonomic efferent and neurochemical expressions of the pulmonary vasculature has been described in a few studies. In human infants and children, a trend towards an increase in the density of nerves can be seen in the arteries of the respiratory unit. In pulmonary hypertensive infants, pre-capillary vessels are characterized by a predominant expression of vasoconstrictor nerves, which may explain their susceptibility to the pulmonary hypertensive crisis [14]. The extent of innervation density from the extrapulmonary vessels to the periphery varies from species to species, and has previously been reported in sheep, pigs and other large animals, and humans [13]. 

Animal use in translational experimental studies is key considering the animal size, anatomy, and physiological characteristics, especially when the results need to be extrapolated in the clinical practice [15]. Pigs and sheep are large animals that share a similar heart structure with humans [16,17]. However, any experimental animal cannot totally simulate human particularities. For cardiovascular interventional procedures, instrument manipulation might be challenging considering the heart position in mammals [18]. For the PADN procedure, nerve structures surrounding the PA are considered as targets [19], thus PA histological characteristics are certainly relevant. We cannot exclude those differences in innervation that might be related to the weight and age of specimens. Thus, sheep and pigs used in our experiments had a significantly lesser weight than patients in whom histological preparations were evaluated. 

Our results are in agreement with a previous report demonstrating PA nerve disposition in pigs aged 2–6 months in the adventitia and up to 2/3 of the outer tunica media [20]. Similar data where nerve fibers were located in the outer third of the tunica media in the PA of sheep and cats were reported by Garland et al. [21] and Knight et al. [22]. Morphometric characteristics, like nerve distribution density, diameter, and distance to the intima in sheep and humans are different; however, these characteristics were similar among mammals. Therefore, it could be assumed that sheep, as well as pigs, might be an appropriate model for PADN procedures. 

By far radiofrequency energy has been extensively employed and studied for denervation procedures [3,4,5,7], although several technical aspects related to device catheters and power settings are under continuous evaluation. Laser energy was introduced in medicine several years ago. However, its role in denervation procedures has not been widely studied. Radiofrequency and laser energy both produce thermal tissue damage by heat, though their mechanism highly differs. Morphological lesion characteristics varied from brown spots and rough defects, and no visible lesions were observed in the PAs when using laser energy in our experiments. Despite this fact, during microscopic analysis in all PA slices, histological changes were found, concluding that lesion macroscopic visualization might not be mandatory. Unlike lesions observed in other ablation procedures where focal necrosis is evident [23], we suggest that artery vasculature features, such as tissue structure, blood flow velocity, plus factors, such as flow irrigation during laser application, might influence lesion formation. Moreover, power energy settings are critical for optimal denervation. Another important aspects that should be taken into account during PADN is the definition of a successful laser session. Difficulties during catheter manipulation that could lead to unsuccessful energy delivery were observed, different to other procedures in which a catheter with electrodes is able to provide electrogram data. The PA might not provide the same references as those seen in the atrium or ventricles. In addition, the use of fluoroscopy guidance only might not be informative enough. 

Acute PA lesions resulting from laser ablation are similar to the histological changes found after radiofrequency ablation, as described earlier by our group [8]. Nerve fiber expression varied from one sheep to another, regardless of whether it was detected in the adventitia and perivascular fat tissue or remained in the tunica media, a fact that could be assumed due to the amount of energy applied.

Recently, Sagerer-Gerhardt et al. used laser energy for renal denervation in experimental animals, and acute thermal damage in the renal artery wall was described [24]. The results of the lesions’ morphological analysis were similar to our findings. Collateral damage observed in the lung parenchyma post laser PADN using a setup of 30 W and 20 s has similar characteristics to the findings after renal laser denervation [24]. Therefore, our findings are in agreement that laser ablation using 20 W power seems safer.

## 4. Limitations

The major limitation of the study is the use of the non-steerable laser ablation catheter, which precluded the intended perpendicular energy delivery to the PA wall in some applications. Theoretically, this may limit the depth of induced necrosis. However, in a recent study, where a similar catheter was used for renal denervation, the orientation of the ablation catheter was less important in the characteristics of lesion formation [24]. In addition, a difference in human and sheep nerve distributions is a limitation that could preclude direct extrapolation of the results.

For neural immunolabeling, we used S100 as the most commonly used marker of nerve fibers and ganglia. S100 is not fully specific for neural tissue and can be found on neurons, Schwann cells, melanocytes, glial cells, myoepithelial cells, adipocytes, Langerhans cells, dendritic cells, and chondrocytes. Following hematoxylin staining, S100 was only used an additional marker for visual assessment of the nerves by two highly experienced pathologists (EBM and EGK). We did not implement other well-known neural markers, such as PGP9.5, which is also not fully specific for the neural tissue; nor tyrosine hydroxylase, since it is specific for sympathetic neural structures only.

## 5. Materials and Methods

The experiment was divided into two phases. In the first phase, we compared pulmonary artery perivascular and vascular nerve distributions between ovine, porcine, and human PA specimens. In the second phase, we performed laser PADN in sheep. A total of 17 experimental animals were included. Seven pigs and 7 sheep underwent PA sample collection, and 3 sheep underwent PA laser denervation. Human PA autopsy material was obtained from 7 subjects (Table 6). All experimental procedures were performed in the facilities of the Preclinical and Translational Research Centre of the Almazov National Medical Research Centre. The Institutional Animal Care and Use Committee-IACUC (protocol 20-15PZ#V2/19-12PZ#V1) reviewed and approved the experimental studies. The human autopsy study was reviewed and approved by the Almazov National Research Centre ethics committee.

### 5.1. Ovine, Porcine, and Human Pulmonary Artery Nerve Distribution Evaluation

PA samples were obtained from 7 male Landrace pigs (mean weight 36.1 ± 2.3 kg) and 7 Katumsky sheep (mean weight 34.4 ± 2.8 kg; *p* > 0.05). Animals were euthanized under general anesthesia by a lethal bolus of potassium chloride as described elsewhere [8]. After sternum removal, gross PA samples were excised and included the following parts: the main PA trunk, PA bifurcation, left and right PAs with the surrounding perivascular tissue, until sub-segmentary branches.

Human PA preparations were obtained from patients who died of non-cardiovascular conditions; none of them had a history of pulmonary hypertension. The patients’ mean age was 64.2 ± 7.1 years with a mean weight of 82.4 ± 5.8 kg. PA samples were excised starting from the pulmonary valve and until sub-segmentary branches, and included perivascular fat tissue. The animal and human samples were transversely cut in 1-cm steps and underwent histological and immunohistochemical examination. A total of 52 histological preparations were made from 28 paraffin blocks in each study group. Sections with a thickness of 2 μm were produced using the Leica RM2235 (Leica, Mannheim, Germany) rotary microtome. All fragments were fixed in 10% buffered formalin, using the standard method; hematoxylin and eosin were used for micro-preparations’ staining, and immunolabeling was performed using anti-S100 protein (DakoCytomation, Glostrup, Denmark). Material processing for the immunohistochemical analysis was previously described in detail [8,25]. The image analyzer Leica Application Suite V 4.5.0 and Leica Scope (Leica, Germany) were used for morphometric analysis. The nerve diameter was calculated for every detected fiber. The number of nerve fibers per cm^2^ characterized the nerve density. The depth of the nerve location was calculated from the endothelial lumen. The above-described PA innervation characteristics were clustered for the following segments: the PA trunk, bifurcation, and right and left PAs.

### 5.2. Ovine Pulmonary Artery Laser Denervation and Histological Evaluation

Three Katumsky sheep (body weight 35–45 Kg) underwent PADN under general anesthesia with intubation (intramuscularly Tiletamine and Zolazepam, 4–7 mg/kg for anesthesia induction; then Isoflurane 100%, 1.5–2%, with O_2_ insufflation through the endotracheal tube at a rate of 7 mL/min). A gastric tube was inserted to prevent aspiration. Preoperative administration of subcutaneous 5000 U heparin was delivered to reduce the risk of thrombosis. Electrocardiographic and invasive systemic blood pressure controls were initiated at the beginning and continued throughout the procedure. A 7F vascular sheath (AVANTI^®^+, Cordis, Florida, USA) was placed into the right femoral artery for systemic arterial pressure monitoring and a steerable introducer (Agilis™ NxT, St.Jude Medical, Minnesota, USA) was introduced via the right femoral vein for PA catheterization. The activated clotting time was controlled every 20 min and kept at >300 s (iStat system, Abbott, Princeton, NJ, USA) by further boluses of intravenous heparin. PA angiography was performed using a multipurpose angiographic catheter (Multipurpose, Cordis, NJ, USA). A laser generator (Medilas D, Dornier Medtech, Wessling, Germany) and a non-steerable open-irrigated laser catheter (RhythmoLas, LasCor GmbH, Taufkirchen, Germany) were used for the PADN procedure. Ablation was performed with a continuous wavelength laser of 1064 nm. The ablation catheter was navigated through the steerable sheath under fluoroscopic guidance in multiple projections (BV Endura C-Arm, Philips, Veenpluis, I Netherlands), and each application was aimed to be delivered as perpendicular to the PA wall as possible. However, due to the rigidity and poor maneuverability of the catheter, some applications were delivered at angles of 45–30°. Laser applications in the PA trunk, its bifurcation and the right and left PAs were delivered using power 10–30 W, with a duration of 10–35 s, and irrigation 40 mL/min. 

Experimental animals were euthanized using the same protocol, and PA preparations were excised as in phase 1 regardless of the presence of evident lesions. Direct visual examination of the PA trunk and left and right PAs allowed identification of areas where ablation was performed. The PA preparations were fixed in 10% buffered formalin, then cut longitudinally. Every detected ablation spot was fully excised (5–10 mm in diameter), then were transversely cut by 1 mm, and micropreparations were stained with hematoxylin and eosin, and immunolabeled to S100.

### 5.3. Statistical Analysis

The results are reported as mean ± SD, absolute numbers, and percentages. Mean values were compared using the unpaired *t*-Student test and ANOVA as appropriate. The differences were considered significant with a *p*-value < 0.05. Statistical analysis was performed using the IBM SPSS Statistics program (version 19.0.0, NY, USA).

## 6. Conclusions

The use of sheep as a model for transcatheter PADN procedures is feasible. Ovine nerve distribution in the PA bifurcation and main branches has specific differences from human PA innervation. Nerve density in the ovine PA is similar to porcine vascular innervation. Laser ablation can be safely used for PADN procedures. The physiological effects of laser PA ablation require further research.

## Figures and Tables

**Table 1:** Comparative morphometric analysis of pulmonary artery nerve fibers and their distribution in pigs, sheep, and humans.

Parameter	Nerve Variable	Pig	Sheep	Human	Pair Comparison (*p*)		
					Pig-Human	Sheep-Human	Pig-Sheep
PA Trunk	Layer innervation	Perivascular adipose, adventitia	Perivascular adipose, adventitia	Perivascular adipose	-	-	-
	Nerve diameter	35.12 ± 4.27	45.43 ± 6.28	118.97 ± 10.38	<0.001	0.001	NS
	Nerve distribution density, Units/cm^2^	48.07 ± 6.34	52.15 ± 11.54	11.66 ± 4.35	0.002	0.013	NS
	Mean depth of nerves from the intima	1541.02 ± 118.49	1785.84 ± 104.3	2404.68 ± 613.06	NS	NS	NS
PA Bifurcation	Layer innervation	Perivascular adipose, adventitia	Perivascular adipose, adventitia, media	Perivascular adipose	-	-	-
	Nerve diameter	31.82 ± 5.83	45.33 ± 8.15	139.18 ± 30.89	0.011	0.022	NS
	Nerve distribution density, Units/cm^2^	91.59 ± 26.74	58.57 ± 8.41	23.49 ± 6.01	0.042	0.012	NS
	Mean depth of nerves from the intima	1338.92 ± 175.83	1515.32 ± 131.69	3126.19 ± 664.07	0.035	0.049	NS
Right PA	Layer innervation	Perivascular adipose, adventitia	Perivascular adipose, adventitia, media	Perivascular adipose	-	-	-
	Nerve diameter	41.36 ± 8.55	39.4 ± 6.66	140.84 ± 9.01	<0.001	<0.001	NS
	Nerve distribution density, Units/cm^2^	63.5 ± 11.36	64.55 ± 7.05	18.04 ± 4.34	0.007	0.001	NS
	Mean depth of nerves from the intima	1059.39 ± 168.45	780.5 ± 140.51	3605.4 ± 1021.56	0.07	0.03	NS
Left PA	Layer innervation	Perivascular adipose, adventitia	Perivascular adipose, adventitia, media	Perivascular adipose	-	-	-
	Nerve diameter	55.94 ± 4.76	43.17 ± 5.59	132.61 ± 9.98	0.002	<0.001	NS
	Nerve distribution density, Units/cm^2^	52.9 ± 30.54	57.47 ± 7.65	18.49 ± 6.28	NS	0.004	NS
	Mean depth of nerves from the intima	1277.12 ± 158.76	916.24 ± 121.93	2268.99 ± 342.85	NS	0.006	NS

NS = non-significant; PA = Pulmonary artery.

**Table 2:** Characteristics of laser applications.

Parameter	PA Trunk		PA Bifurcation		Left PA		Right PA	
	Laser Setup (W–Time)	Ablation Sessions	Laser Setup (W–Time)	Ablation Sessions	Laser Setup (W–Time)	Ablation Sessions	Laser Setup (W–Time)	Ablation Sessions
Sheep №1	20 W, 20 s	6	20 W, 20 s	2	10 W, 30 s	4	15 W, 20 s	5
Sheep №2	20 W, 25 s	3	20 W, 25 s	3	30 W, 20 s	2	30 W, 20 s	3
Sheep №3	20 W, 35 s	3	20 W, 35 s	2	20 W, 35 s	5	20 W, 35 s	4

PA = Pulmonary artery; s = seconds; W = Watt.

**Table 3:** Semiquantitative assessment of ablation lesions.

Grade	Histology Findings	Comment
Grade III	Hemorrhages at any depth of the media, in the adventitia and adipose tissue (with a diameter more than 800 µm). Dissections of the artery wall with a depth of more than 50% of the media thickness. Coagulation necrosis of perivascular adventitia and adipose tissue with/without damage to nerve fibers.	A total transmural lesion that involves all possible neural structures within the artery wall and/or in the adventitia.
Grade II	Focal hemorrhages at any depth of the media, in the adventitia and adipose tissue (with a diameter of 200–800 µm). Focal necrosis of the intima and the inner third of the media and (depth up to 10% of the thickness of the media layer). Absence of damage in the form of necrosis in the adventitia and adipose tissue. Dissections of the artery wall with a depth of up to 50% of the media thickness.	A non-transmural lesion that involves part of neural structures within the artery wall or in the adventitia.
Grade I	Focal edema of the inner third of the media and extending to a depth of up to 10% of the PA wall thickness. Small-focal hemorrhages at any depth of the media and in the adventitia (up to 200 µm in diameter). Absence of damage in the form of necrosis in the adventitia and adipose tissue.	A PA wall lesion without any evidence of nerve damage.

Μm = micrometer.

**Table 4:** Grade lesion according to the power employed for pulmonary artery laser denervation.

Number of Lesions	Grade	Power
1	III	10 W
1	III	15 W
8	I	20 W
7	III	20 W
6	II	20 W
1	III	30 W
1	II	30 W
Total: 25	I-8 II-7 III-10	20/30 W 20/30 W 10/15/20/30 W

**Table 5:** Semiquantitative assessment of ablation safety.

Parameter	Histology Findings	Power Setting
Safe	No evidence of any collateral damage. PA wall dissection is seen only microscopically, if present.	≤20 W
Unsafe	Clear evidence of extravascular damage: hemorrhages in the lungs, atrial of the ventricular myocardium. Macroscopically severe PA wall dissection.	20 W and 30 W

PA = Pulmonary artery.

**Table 6:** Patient characteristics, cardiovascular history, and medications.

№	Sex	Age	Disease	Cause of Death	Concomitant Cardiovascular Disease	Medications
1	M	60	Small cell lung cancer	Hemorrhage	Arterial hypertension	ACEi, diuretic, BB
2	M	69	Large cell neuroendocrine lung cancer	Paracancrotic pneumonia	Arterial hypertension	Diuretic, ARB, Antibiotics
3	M	70	Brain glioblastoma	Hemorrhagic cerebral infarction	Arterial hypertension	Dexamethasone, LMWH, ACEi, diuretic, CCB
4	M	62	Brain glioblastoma	Hemorrhage	Atherosclerosis	Dexamethasone, LMWH,
5	M	49	Pancreatic neuroendocrine tumor	Peritonitis	No	Antibiotics
6	M	65	Colon adenocarcinoma	Hemorrhage	Arterial hypertension	Diuretic, Heparin
7	M	61	Brain glioblastoma	Cerebral edema	No	Dexamethasone

ACEi = Angiotensin-converting enzyme inhibitor; ARB = Angiotensin II receptor blocker, BB = beta-blocker; CCB = calcium channel blocker; LMWH = Low-molecular-weight heparin; M = male.

## Data Availability

Data related to this study can be provided by the corresponding authors on request.
